# Pulmonary hypertension and insulin resistance: a mechanistic overview

**DOI:** 10.3389/fendo.2023.1283233

**Published:** 2024-01-04

**Authors:** Tamires M. Zanotto, Any Elisa de Souza Schmidt Gonçalves, Mario J. A. Saad

**Affiliations:** ^1^ Department of Internal Medicine, State University of Campinas (UNICAMP), Campinas, SP, Brazil; ^2^ Departament of Medical Clinics, Obesity and Comorbidities Research Centre (O.C.R.C.), State University of Campinas (UNICAMP), Campinas, SP, Brazil

**Keywords:** pulmonary arterial hypertension, insulin resistance, oxidative stress, endoplasmic reticulum stress, mitochondria dysfunction

## Abstract

Pulmonary arterial hypertension (PAH) is a vascular remodeling disease, characterized by increased blood pressure levels in pulmonary circulation, leading to a restriction in the circulation flow and heart failure. Although the emergence of new PAH therapies has increased survival rates, this disease still has a high mortality and patients that receive diagnosis die within a few years. The pathogenesis of PAH involves multiple pathways, with a complex interaction of local and distant cytokines, hormones, growth factors, and transcription factors, leading to an inflammation that changes the vascular anatomy in PAH patients. These abnormalities involve more than just the lungs, but also other organs, and between these affected organs there are different metabolic dysfunctions implied. Recently, several publications demonstrated in PAH patients a disturbance in glucose metabolism, demonstrated by higher levels of glucose, insulin, and lipids in those patients. It is possible that a common molecular mechanism can have a significant role in this connection. In this regard, this narrative review intends to focus on the recent papers that mainly discuss the molecular determinants between insulin resistance (IR) associated PAH, which included obesity subclinical inflammation induced IR, PPAR gamma and Adiponectin, BMPR2, mitochondrial dysfunction and endoplasmic reticulum stress. Therefore, the following review will summarize some of the existing data for IR associated PAH, focusing on the better understanding of PAH molecular mechanisms, for the development of new translational therapies.

## 1 Introduction

Pulmonary hypertension (PH) is defined by a mean pulmonary arterial (PA) pressure higher than 20 mm Hg at rest, measured by right heart catheterization. The symptoms of PH include fatigue, inability to exercise, and dizziness. However, the delay in recognizing the beginning of symptoms in PH patients worsens the prognosis, leading to a severe vascular disfunction and right ventricular (RV) failure (1, 2).

The different forms of PH are categorized into five clinical groups: 1- PAH (pulmonary artery hypertension) which includes heritable, idiopathic, drug-induced, PAH associated with connective tissue disease, HIV, and portal hypertension; 2- PH due to left-sided heart disease; 3- PH due to lung disease, hypoxia or both; 4- PH due to pulmonary artery obstruction; and 5- PH with multifactorial or unclear mechanisms, which include hematological and systemic and metabolic disorders (3).

PAH, or clinical group 1 is a relatively rare disease, classically being a result of the obstructive vascular remodeling due to the increase in PA pressure. Moreover, in some acute conditions, such as PA pressure elevation under hypoxia or high-altitude exposure, there is a vasoconstriction that also contributes to PAH development Lastly, many other factors play a role in PAH development, such as the release of inflammatory cytokines, growth factors, and hormones in blood vessels, and this mechanism needs to be further elucidated (4, 5).

The incidence and prevalence are not completely known, and a French study suggests that fifteen persons per million are affected with PAH, and the incidence is higher in females. It was shown that in the incident cohort, there was an 88% one-year survival (6, 7).

In addition to the gender component, some risk factors are being recognized for this disease, such as obesity, the use of some appetite suppressant drugs, hormone therapy, patients with associated insulin resistance (IR) and others (8–10).

Obesity leads to an increase in PH incidence and severity, being associated to hypoxia, due to the higher mechanical load of fat in obese patients and metabolic abnormalities, due to its characteristic of chronic low-grade inflammation, which is being implied as causal for PH (11). However, there is an obesity paradox, wherein obese patients had a lower mortality, which was described in cardiopulmonary diseases, including PAH. In a seminal work, which assessed the French PAH Network registry, it was shown that even obesity being not associated to increased mortality in PH, there was an age-obesity interaction increased mortality, among young patients that presented increased mortality, showing the importance of active weight management in younger PH obese patients (12).

In the progress of PAH there is a disorganized endothelial function, with changes in the vascular tone, and an aberrant vascular cell remodeling, with an accumulation of inflammatory cells in the lumen of the pulmonary arteries, leading to an increase in pulmonary vascular resistance and RV hypertrophy, with consequent heart failure. This PAH associated inflammation includes abnormalities that are not only restricted to the pulmonary arteries, but also extended to different organs that share a common mitochondrial abnormality, leading to mitochondrial dysfunction (13).

In this narrative review, we aimed to further explore this process and other inflammatory metabolic dysfunctions, such as endoplasmic reticulum (ER) stress, and especially IR to better understand its correlation to PH (4, 13–16).

Moreover, regarding the different types of treatment for PH, none of them can effectively reverse the disease or significantly improve the long- term survival. Eventually, lung or lung and heart transplantation is the best option for patients who continue to progress the symptoms (17). However, in recent years important progress in epidemiology and molecular mechanisms has opened new avenues for treatment.

## 2 Correlation of IR with PAH

The correlations between obesity, IR, dyslipidemia and cardiovascular disease are well established. However, the link between these diseases and PAH development has only recently been explored (18).

As we know, obesity is a major public health issue and a well-recognized risk factor for IR. More recently, evidence has emerged for the pathogenic role of obesity in the development of PH. Studies have shown an increased prevalence of hypertension in the pulmonary circulation of obese individuals compared to lean controls. Nonetheless, the idiopathic form of PAH is more prevalent in obese individuals (19–21).

IR was demonstrated to be significantly more prevalent in PAH female patients than in the general population, and this might affect morbidity in those PH patients. Moreover, PH seems to occur more often in postmenopausal and obese females (22).

Patients with idiopathic PAH showed reduced glucose tolerance, and this was correlated with a reduced response to hyperglycemia in PAH, but normal insulin secretion during hyperglycemic clamps analyses. Unexpectedly, the insulin sensitivity in muscle was improved in these patients, but the hepatic insulin extraction was increased, being a possible underlying factor. Metabolomics of this cohort showed that lipid oxidation and ketones were increased in PAH (23).

Human studies suggest that IR in obese is accompanied by elevations in circulating inflammatory cytokines, endothelial dysfunction and mitochondrial dysfunction, that are commonly associated with PAH. Hemoglobin HbA1c, used as a sensitivity test for diabetes mellitus (DM), was increased in PAH patients, with an increased glucose intolerance and an elevated risk for DM development in those patients (24).

PAH patients with associated IR showed worse functional class and diastology, with unfavorable parameters of left ventricular diastolic function, including mitral inflow, compared to insulin sensitive PAH patients (25).

For the right ventricle, PAH patients with DM had a lower RV stroke work index (RVSWI), a measurement of RV workload, compared to PAH patients with no DM. Among the PAH patients with DM, patients who died had significant decreased RVSWI than survivors, and the survival rate was 10 years lower, compared to PAH patients without DM (26).

Several publications have shown in PAH patients a disturbance in glucose metabolism associated with IR, demonstrated by higher levels of glucose, insulin, and lipids in those patients (14, 23, 25, 27–29).

In summary, there is a clear association between PAH and IR. It is possible that common molecular mechanisms can have a significant role in this connection. In this regard we will further explore these molecular mechanisms that can integrate PAH and IR, which include subclinical inflammation, peroxisome proliferator-activated receptor gamma (PPARg) and adiponectin, bone morphogenic protein receptor type 2 (BMPR2), mitochondrial dysfunction, and ER stress.

## 3 A common ground: molecular determinants of the association between IR-PAH

### 3.1 Subclinical inflammation

**Figure 1:**
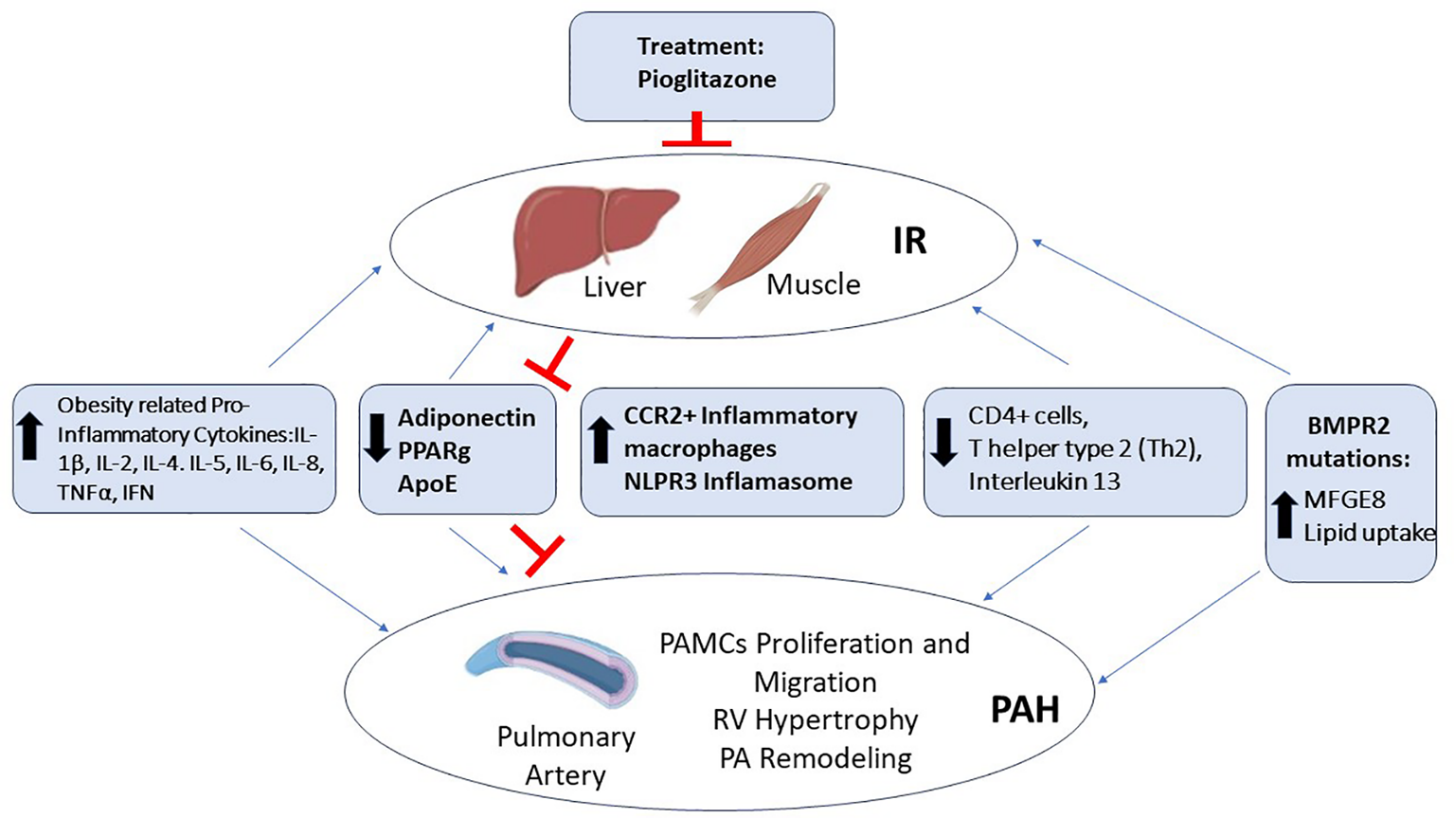
Molecular determinants of the association between IR-PAH. Several factors are implicated in PAH patients with IR, among them are the increased levels of obesity related cytokines, such as Interleukin 1β (IL-1β), Interleukin 2 (Il-2), Interleukin 4 (IL-4), Interleukin 5 (IL-5), Interleukin 6 (IL-6), Interleukin 8 (IL-8), Tumor Necrosis Factor α (TNFα), and Interferon (IFN); increased inflammation through the nucleotide-binding domain, leucine-rich–containing family, and pyrin domain–containing-3 (NLPR3) Inflamasome and monocyte-derived C-C chemokine receptor 2 (CCR2+) macrophages; and bone morphogenic protein receptor type 2 (BMPR2) mutations leading to increased lipid uptake. In contrast, there is the obesity related decreased levels of the hormone Adiponectin and its target proliferator-activated receptor gamma (PPARg); the decreased levels of Apolipoprotein E (ApoE), immune CD4+ lymphocytes (Th2), and Interleukin 13 (IL-13), leading to IR associated PAH. Treatment with Roziglitazone and Pioglitazone are able to improve the levels of Adiponectin and PPARg, blocking this IR associated PAH.

At the level of the pulmonary artery (PA), the occurrence of inflammation is a well demonstrated phenomenon in PAH development, also related to obesity and IR (**Figure 1**).

PAH overweight and obese patients had worse health-related quality of life and increased incidence of hospitalizations compared to normal weight, showing the importance of weight maintenance despite decreased transplant-free survival (30).

PAH in obese patients was believed to be consequence of hypoventilation and hypoxia, due to the higher mechanical load of excess body fat. However, studies are now implying that the inflammatory set of obesity may also have a causal role for the development of PH. After treatment with a high-fat diet (HFD), obese rats were assessed and showed an increase in PA remodeling and PA pressure, with a neomuscularization of distal arterioles and RV hypertrophy (RVH). They also showed increased levels of circulating cytokines, and hyperlipidemia in the PA wall of this model, with no evidence of hypoventilation, confirming that obesity associated inflammation can lead to PH, without hypoxia (31).

PAH is implicated with inflammasome activation; the inflammasomes are protein complexes that respond to infections and inflammatory stimuli, propagating a signaling of danger in affected tissues. Inflammasome products, such as interleukins and cytokines have been described as biomarkers of PAH, and are possible targets for treatment of this disease (32). Patients with PAH showed an increase in several serum cytokines such as Interleukin 1β, Interleukin 2, Interleukin 4, Interleukin 5, Interleukin 6, Interleukin 8, Tumor Necrosis Factor α (TNFα), and Interferon (IFN), and these levels had a previously unrecognized impact in patient survival (33).

PA remodeling in PAH occurs after an increase in vascular smooth muscle cells (SMCs) density, and studies have suggested an immune pathogenesis in the development of this lesion. The use of an antigen (*Aspergillus fumigatus*) in mice, through the airways, induced severe muscularization in small or medium-sized PA. After depletion of CD4+ lymphocytes, antigen-specific T helper type 2 (Th2), and the pathogenic interleukin 13, there was a decrease in PA muscularization. Moreover, the increase in PA muscularization was also associated with an increase in epithelial cells and macrophages, implying that the innate and adaptive immune response has a role in PAH (34).

In PAH patients with idiopathic or associated PAH, there is also the formation of plexiform lesions, which are a vascular glomerular structure, forming channels in branches of the PA. These lesions occur with a nonregulated growth of modified SMCs, which expresses abnormal growth factors, such as the growth factor VII-related antigen and vimedin, a growth differentiation-related intermediate filament. There is also an implication in the formation of plexiform lesions related to a perivascular inflammatory cell infiltration, in most of the cases composed by the presence of T and B cells, and macrophages in endothelial cells (35).

As mentioned previously, the development of PAH in patients comes even in the absence of obesity associated inflammation and diabetes, however, PAH patients with obesity associated IR have a worse prognosis compared to PAH patients that do not have obesity, showing the importance of further investigation of obesity-induced IR and its association to PAH (25, 29, 36).

It’s possible that the inflammation described in obesity can also have a role to RV failure in PH. In a rat model of decompensate RVH (monocrotaline [MCT] and Sugen-5416 hypoxia [SuHx]), increased levels of macrophages, fibrosis and natriuretic peptides in the RV were demonstrated. The monocyte-derived C-C chemokine receptor 2 (CCR2+) inflammatory macrophages were specifically increased, with high expression of the nucleotide-binding domain, leucine-rich–containing family, and pyrin domain–containing-3 (NLPR3) inflammasome, leading to mitochondrial dysfunction. In contrast, inhibition of NLPR3 improved the RV function in these mice (37).

### 3.2 PPARg and adiponectin

Data coming from different sources demonstrated a reduction in expression of the PPARg in PAH patients (18, 38, 39). PPARg is a nuclear receptor and transcription factor that regulates adipogenesis and glucose metabolism. PPARg deficiency was pointed out as a trigger for IR.

PAH patients have decreased levels of apolipoprotein E (ApoE), a factor that reduces circulating low-density lipoprotein (LDL), and consequently, atherogenesis, and decreased levels of PPARg in the lungs, and this was associated with decreased levels of adiponectin, leading to IR. Moreover, ApoE deficiency leads to increased platelet-derived growth factor-BB (PDGF-BB)/mitogen-activated protein kinase, a factor that induces SMCs proliferation and migration, leading to PAH. Therefore, ApoE, PPARg, and adiponectin decreased levels can induce IR and contribute to PAH pathogenesis (40).

ApoE knockout mice treated with an HFD demonstrated to have severe PH and IR. Treatment with the agonist of PPARg (Roziglitazone), an insulin sensitizer and antidiabetic drug, can reverse the PH (40) in those mice. In this regard, treatment with Pioglitazone (PIO), another agonist of PPARg, was also capable of improving IR and PH in mice treated with an HFD, with an increase in adiponectin levels (18).

In a work performed by our group, we investigated the effects of PIO in obese mice treated with an HFD, with confirmed IR and PH. The results showed that the HFD was able to increase PA pressure. We also demonstrated a reduction in circulating adiponectin, and perivascular adiponectin expression in the PA of these mice, with a reduction of PPARg expression. The treatment with PIO was capable of improving this IR and reduced PH, by reversing the decreased expression of adiponectin and PPARg, highlighting the mechanisms involved in PAH patients with IR in obesity, and the potential use of PIO as a treatment (18).

Deletion of PPARg in cardiomyocytes can cause biventricular systolic dysfunction and intramyocellular lipid accumulation in mice. In a mouse model of hypoxia, treatment with PPARg agonist PIO was capable of reversing severe PAH, preventing RV failure. Furthermore, PPARg activation was demonstrated to normalize epigenetic and transcriptional regulation related to disturbed lipid metabolism, besides mitochondrial function, showing the importance of this receptor as a therapeutic target (41).

Moreover, the decrease in PPARg levels are also being associated with changes in its target hormone adiponectin. Adiponectin is an adipokine, also known to be a homeostatic factor, through the regulation of lipid and glucose metabolism, having an anti-inflammatory role that improves insulin sensitivity (42, 43).

It seems that adiponectin has a protective role in metabolic and pulmonary vascular disease, because of its vasodilator activity, and its ability to suppresses vascular inflammation (44, 45). It was demonstrated that this hormone is localized in the luminal side of endothelial cells in lungs, blocking inflammatory TNF in those cells. Moreover, gene deletion of adiponectin in mice was capable of increasing inflammatory cell infiltration and PA pressure (46).

Different studies demonstrated that adiponectin suppresses SMCs proliferation and migration. Moreover, the effect of decreased levels of adiponectin in PAH patients is also being implied in tissue remodeling. There is also evidence that adiponectin protects against myocardial ischemia-reperfusion injury through AMPK and COX-2 dependent mechanisms (47–49).

In summary, decreased levels of PPARg and adiponectin in PAH patients is being pointed out as an important mechanism for the worsening of PAH, which needs to be further explored as a target for PAH treatment. This occurs through different mechanisms, such as the induction of SMCs proliferation and migration leading to an increase in PA pressure, and also adiponectin can suppress inflammatory reactions in myocardium.

### 3.3 BMPR2

The metabolic defects of obesity and IR were also implicated with the transforming growth factor-β (TGF-β) family receptor, called BMPR2, which is a serine/threonine kinase involved in different cellular functions, including the growth and differentiation of SMCs and endothelial cells, being important in vasculogenesis (50). Mutations in BMPR2 structure leads to a decrease in BMPR2 expression and are present in approximately 70% of patients with familial PAH and 20% of patients with sporadic idiopathic PAH, being implied with the initiation and progression of PAH, involved in the regulation of vascular remodeling and inflammation in the lung (50–52).

In an HFD fed mice model of IR, with inducible expression of BMPR2 mutation (BMPR2R899X), an increase in body weight through fat accumulation in skeletal muscle, and decreased oxygen consumption were demonstrated. These mice also had strong increases in PH. This data indicated that this IR occurred in an early feature of BMPR2 mutation, associated with skeletal muscle pathology and that this precedes the emergence and worsening of PAH, being a possible causal link between obesity and PAH (53).

Patients with mutation in BMPR2 that develop PAH demonstrated clear alterations in lipids metabolism. BMPR2 mutant cardiomyocytes have decreased mitochondrial respiration and increased mitochondrial superoxide production. These mutant cells showed an increase in milk fat globule-EGF factor-8 protein, which impairs the insulin signaling via protein kinase B (Akt). BMPR2 mutation was also implied to enhance lipid uptake, leading to RV lipotoxicity (51). In this manner, PAH patients demonstrated higher levels of lipoprotein axis-related IR, and oxidized LDL lipoprotein accumulation within macrophages in lungs, showing that these patients have alterations in the lipid and lipoprotein axis (28).

Rats with a monoallelic deletion of 71bp (Δ*71* rats) in exon 1 showed decreased BMPR2 expression, developing age dependent spontaneous PAH with a low penetrance, similar to humans, exhibiting a progressive pulmonary vascular remodeling with a proliferative phenotype (54).

Mice with specific deletion of BMPR2 in endothelial cells develop hypoxia-induced PAH, with decreased lung tumor protein p53 (EC p53), peroxisome proliferator-activated receptor gamma coactivator 1-alpha (PGC1α), and transcription factor A (TFAM) regulators of mitochondrial biogenesis and DNA repair, leading to mitochondrial dysfunction (55). In this regard, human endothelial cells with mutations in BMPR2 kinase domain (BMPR2R332X) or in the cytoplasmic tail domain (BMPR22580delT) were correlated to increased insulin levels in plasma, with an impairment in insulin-mediated glucose uptake, through reduced glucose transporter translocation (56).

These studies suggested that mutations in BMPR2 lead to mitochondrial dysfunction and hyperinsulinemia in IR-PAH progress and that this phenomenon is the result of different mechanisms, such as the occurrence of a lipid accumulation in skeletal muscle and the RV, and an impairment in mitochondrial homeostasis including the PA, with decreased mitochondrial respiration and DNA repair.

The functional and molecular link between loss of BMPR2 in PA smooth muscle cells (PASMCs) is also important for understanding PAH pathogenesis and was further investigated in different human and mouse models.

PASMCs specific BMPR*^−/−^* transgenic mice *(BKO^SMC^)* showed decreased hypoxia-induced vasoconstriction and persistent PH, following recovery of hypoxia, with sustained muscularization of distal PA. PASMCs from these mice also had reduced contractility, increased proliferation, and apoptosis resistance. The reduction of BMPR2 in human PASMCs, using small interference RNA, and human PASMCs from PAH patients with BMPR2 mutations showed a similar phenotype, with upregulation of pERK1/2 (phosphorylated extracellular signal related kinase 1/2)-pP38-pSMAD2/3 mediating elevation in ARRB2 (β-arrestin2), pAKT inactivation of GSK3-beta, CTNNB1 (β-catenin) nuclear translocation, and reduction in RHOA (Ras homolog family member A) and RAC1 (Ras-related C3 botulinum toxin substrate). Decreasing ARRB2 in PASMCs with reduced BMPR2 restored normal signaling and reversed the impaired contractility and proliferation in those cells. These results highlight that agents that neutralize the increased ARRB2 in PASMCs with loss of BMPR2, could be a therapeutic target for PAH (57).

It was demonstrated that PAH patients, who are carriers of BMPR2 mutations, are younger at diagnosis, with a higher mean PA pressure and pulmonary vascular resistance, being less likely to respond to acute vasodilator testing than the other patients. Regarding survival, these younger patients that presented BMPR2 mutations had increased risk of death, or transplantation compared to those without mutations (57). Additionally, BMPR2 deficiency has been associated with the pathological degree and worse prognosis of PAH. Patients of PAH showed early disease onset by 10 years. Moreover, increased BMPR2 activity leads to a decline in cellular growth and proliferation in the pulmonary vasculature, showing the importance of this protein as a target for PAH treatment (58).

## 4 Mitochondrial dysfunction

**Figure 2:**
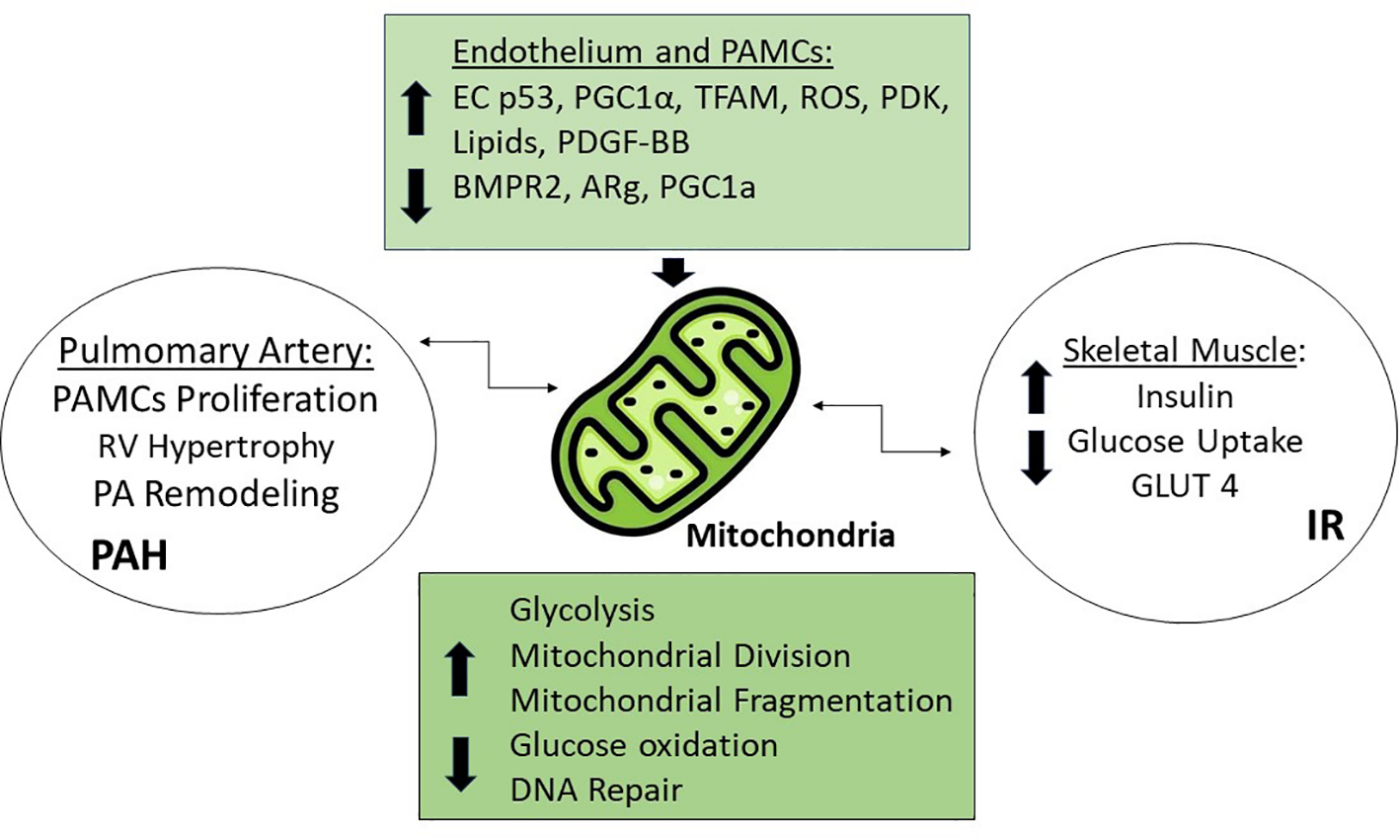
Mitochondrial Dysfunction. Increased levels of Tumor protein p53 (EC p53), Peroxisome proliferator-activated receptor gamma coactivator 1-alpha (PGC1α), Transcriptor Factor A (TFAM), Reactive Oxygen Species (ROS), Pyruvate dehydrogenase kinase (PDK), platelet-derived growth factor BB (PDGF-BB), lipids, and the decreased levels of bone morphogenic protein receptor type 2 (BMPR2), and PPARg coactivator 1a (PGC1a) are responsible for mitochondrial dysfunction through different mechanisms, such as increased glycolysis, mitochondrial division and fragmentation, and decreased glucose oxidation and DNA repair leading to PAH in the pulmonary artery and IR in muscle.

The emerging theory that PAH needs to be seen as a metabolic syndrome, with many organs involved, shares the idea of a common abnormality in mitochondria, the most important energy production site in the body (**Figure 2**). Many studies have shown that the development of PAH is closely related to mitochondrial dysfunction, which includes the tricarboxylic acid cycle, redox homeostasis, enhanced glycolysis, increased ROS expression etc. (13, 59). The hypertrophy in the RV seems to be associated with a decreased mitochondrial glucose oxidation and a glycolytic phenotype (60). In this regard, patients with diabetes mellitus type 2 (DM2) also have an imbalance between glycolytic and oxidative phosphorylation activity in skeletal muscle (61).

The reduction of RV function and electrical remodeling was studied in two rat models of RVH, and was associated with a pyruvate dehydrogenase kinase (PDK) mediated glycolytic mitochondrial shift in the RV. When PDK was inhibited, there was a partial restoration of RV function, and a decrease in RVH, with an RV repolarization and increased glucose oxidation. This mechanism highlights possible targets for RVH (60).

Patients with diabetes have pulmonary vascular complications, and this occurs mainly because glucose is the major energy source for maintaining vascular contraction. Glucose metabolism in blood vessels is mostly related to glucose anaerobic fermentation, and a chronic metabolic disorder in this process can damage and increase the mitochondria division in vascular endothelial, and SMCs, affecting the tone of vessels (59).

There is strong evidence linking the accumulation of lipids in skeletal muscle, leading to IR in PAH patients. This event is also being associated with mitochondrial dysfunction, through suppressed glucose oxidation and upregulation of glycolysis (62).

As mentioned before, PAH is associated with a decrease in PPARg levels, and this stimulates PASMCs proliferation. PPARg seems to also perform an important function in mitochondria, being in association with PGC1α, a regulator of mitochondrial biogenesis and gene expression. After knocking down PPARg or PGC1α in human PASMCs, there is a decrease in mitochondrial mass and an increase in its fragmentation, impairing its function in these cells (63).

Another study showed that the exposure of PASMCs to PDGF-BB, which regulates cellular division, induced changes in mitochondrial morphology, with an increase in ROS production. Treatment of these cells with the glucagon-like peptide-1 receptor agonist liraglutide, a class of drug used in DM2 patients, that mainly acts stimulating insulin secretion, prevented this ROS production. Liraglutide also increased the expression of α-smooth muscle actin and mitigates PASMCs proliferation, through the inhibition of nicotinamide adenine dinucleotide phosphate oxidases pathways in PAH (64). Moreover, treatment with liraglutide was also shown to protect rats with PH induced by monocrotaline, by inhibiting PDGF-BB stimulated PASMCs migration and decreasing levels of endothelial nitric oxide synthase and Rho kinase pathways, having a therapeutic role in pulmonary vascular remodeling (65).

The use of some substances, such as amphetamines (AMPH) or methamphetamines (METH) can cause oxidative stress and mitochondrial dysfunction, being a risk factor for PAH. PAH patients with historical use of AMPH have a role in DNA damage of PA endothelial cells, in hypoxic conditions. This phenomenon involves the activation of protein phosphatase 2A, that can inhibit the Akt pathway, and consequently, lead to IR (66). Furthermore, there is an increase in Sirtiun 1 protein, with consequent degradation of HF1α, decreasing its transcriptional activity and the pyruvate dehydrogenase kinase. Finally, there is an increase in mitochondrial oxidative phosphorylation, resulting in an impaired electron transport and an increase of ROS, leading to caspase-3 activation and DNA damage. Mice treated with METH, in hypoxia states, have HIF1α suppressed and PA DNA damage, with oxidative stress and worsening of vascular remodeling (66).

Nevertheless, the mechanisms implied in mitochondrial dysfunction associated IR and the emergence of PAH, have multiple facets, and need to be further elucidated. The mitochondrial dysfunction in patients with PAH, for example, can be caused by a disruption of the mitochondria ER (67), since the ER releases the calcium needed for the mitochondrial enzymes activation. When the stress is high or chronic, the ER can send signals to the mitochondria to start programmed cell death (apoptosis), showing the importance of this organelle in this process (68). However, abnormal mitochondria in PAH can suppress this event, and increase vascular remodeling and heart failure (59).

## 5 Endoplasmic reticulum stress

**Figure 3:**
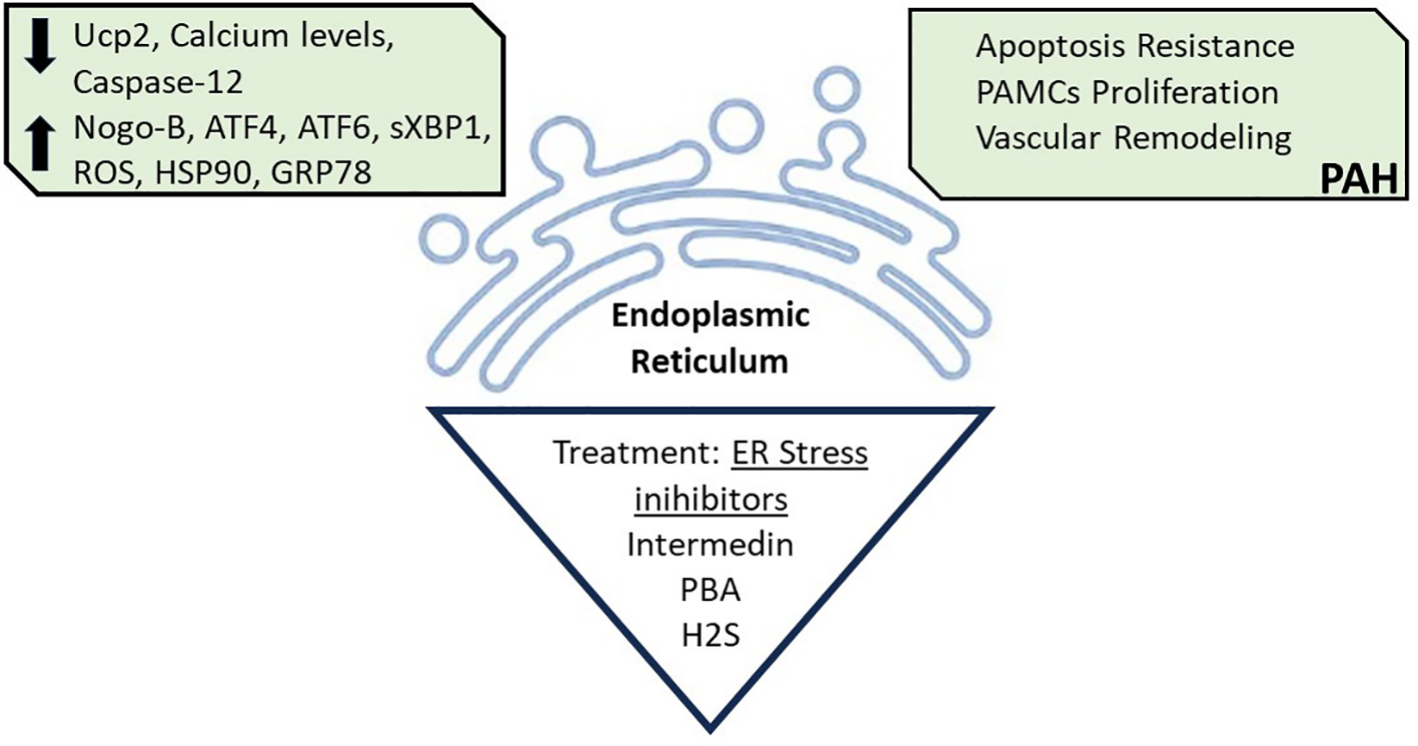
Endoplasmic Reticulum Stress. Endoplasmic reticulum disruption caused by decreased levels of Uncouple protein (Ucp2), Calcium, and Caspase-12; and increased levels of Reticulon 4B (Nogo-B), Transcription factor 4 (ATF4), Transcription factor 6 (ATF6), Spliced form X- box binding protein 1 (sxBP1), Reactive oxygen species (ROS), Heat shock protein 90 (HSP90), and Glucose-regulated protein 78 (GRP78), is associated with PAH development. Treatment with ER stress inhibitors, such as with pharmacological Intermedin, 4-Phenyl butyric (PBA) and hydrogen sulfide (H2S) can reverse the PAH associated ER stress. All figures were created with the website Biorender.com.

The ER is the major site for protein synthesis and processing, and calcium metabolism (**Figure 3**). However, some pathological conditions can disrupt ER homeostasis, leading to ER stress, which is a key mechanism of insulin resistance in liver and muscle of obesity, and is also implied in PAH trigger (69, 70).

The mechanisms by which ER stress can contribute to PAH include an increase in protein reticulon (Nogo-B) in PASMCs as demonstrated in patients with PAH and in experimental models, and also an increase in the molecular chaperone heat shock protein 90 (HSP90) (71–73).

Other conditions were associated to ER stress in PAH patients, such as genetic mutations in BMPR2. It was demonstrated that ER stress chemical inhibitors, such as 4-Phenyl butyric (4-PBA) can rescue expression of mutant-BMPR2 and could have a therapeutical role in those patients (74, 75).

It was also demonstrated that treatment with the peptide Intermedin, an inhibitor of ER stress, in rats under hypoxia, improved PAH by preventing PASMCs hypertrophy, leading to a reduction in wall thickness and an increase in lumen diameter of the PA. This treatment ameliorates PASMCs hypertrophy, through the activation of the ER stress apoptosis pathway, by an increase in the protein caspase-12, decreasing the vascular remodeling in PAH (76).

Another ER stress inhibitor that has a potential role as a therapeutic target for ER-mitochondrial disruption is the anti-inflammatory hydrogen sulfide (H2S). Human PASMCs, after hypoxia, were treated with an H2S donor and this treatment was sufficient to inhibit PASMCs proliferation. *In vivo*, treatment with H2S reversed the hypoxia induced PAH in rats. This occurred due to a suppression of ER stress, with a decrease in ATF-6 protein marker, showing that H2S could be used as a PAH therapy (77).

In cutaneous systemic sclerosis patients with PAH, there is an increase in the expression of ER stress markers, including activating transcription factor 4 (ATF-4) and ATF-6, and the spliced form X- box binding protein 1 (sXBP1) in blood mononuclear cells, suggesting that this associated disease can be affected by ER disruption (78).

The ER stress occurrence can influence insulin signaling, leading to IR (79). In this regard, our group demonstrated the role of inflammatory iNOS in obesity- associated IR and ER stress. We showed in obese mice that the deletion of iNOS was essential for IR improvement in muscle, and partially dependent for IR improvement in liver and adipose tissue after iNOS deletion, with a clear associated ER stress. Furthermore, we demonstrated that chemical inhibition of this ER stress in iNOS KO obese mice, was capable of improving this IR and restoring the glucose tolerance (80).

Following this idea, PAH patients with associated ER stress, could have more chances of worsening the insulin sensitivity.

It was demonstrated in human PASMCs, after ER stress induction, that the glucose-regulated protein 78 (GRP78), a key chaperone in stress conditions was increased, and this increases the expression of inflammatory and ER stress markers. Moreover, the plasma levels of GRP78 were elevated in PAH patients, being a potential biomarker for the mortality risk in these patients (81).

On the other side, in our model of HFD-induced IR accompanied by sustained increased in PH in mice, we did not demonstrate ER stress in pulmonary artery. This data suggests that although in our mice model of obesity associated insulin resistance multiple mechanisms may contribute to induce PH, a disruption in ER homeostasis is not involved.

## 6 Final considerations

Although various molecular determinants of PH are now known and have been described here, some points should be better investigated: First, which molecular determinant correlates better or more implicated in specific forms or different etiologies of PAH. In addition, the link between these molecular determinants is another point that can help the investigation of new therapeutic approaches to PAH. Finally, the effect of drugs that block these mechanisms in human trials is another gap in the area.

The link between IR and PH might have therapeutic implications. Drugs used to treat DM2 and IR have also been investigated in PH. Metformin, the most prescribed drug in patients with DM2, also improves IR. In a recent single-arm, open-label phase II study in patients with PH, metformin improved the RV fractional area and in parallel reduced triglyceride content. These improvements correlated with beneficial changes in lipid and glucose metabolism markers. Sodium glucose linked transporter inhibitors are another class of drugs used to treat DM2, that reduces hyperglycemia by increasing glycosuria, but also improves IR. In a randomized, multicenter, double-blind, placebo-controlled trial, empagliflozin produced rapid reductions in PA pressures, which were amplified over time. Moreover, the most effective drug to treat IR, pioglitazone, shows the most impressive results in improving PAH in animal models (38, 41), in accordance with our results (18). We showed that pioglitazone reverses the reduction in PPARg expression in the PA (18). In addition, Legchenko et al. showed that pioglitazone by activation of the PPARg, restores fatty acid oxidation and protects against heart failure in a rat model of PAH (41). Future human trials would be required to evaluate pioglitazone safety and efficacy in humans.

In summary, our review shows that obesity/IR can induce and aggravate PH, with clear molecular changes in the PA that connect both conditions. Moreover, the molecular mechanism for the association of obesity/IR with PH seems not to be related to ER stress, pointing to a reduction in circulating and perivascular adiponectin, associated with subclinical inflammation that can reduce expression of PPARg in the PA and consequently induce mitochondrial dysfunction.

## Author contributions

TZ: Conceptualization, Investigation, Writing – original draft, Writing – review & editing. AG: Writing – original draft. MS: Funding acquisition, Supervision, Writing – review & editing, Writing – original draft.
